# GC- and AT-rich chromatin domains differ in conformation and histone modification status and are differentially modulated by Rpd3p

**DOI:** 10.1186/gb-2007-8-6-r116

**Published:** 2007-06-18

**Authors:** Job Dekker

**Affiliations:** 1 Program in Gene Function and Expression and Department of Biochemistry and Molecular Pharmacology, University of Massachusetts Medical School, Plantation Street, Worcester, MA 01605-4321, USA

## Abstract

**Background:**

Base-composition varies throughout the genome and is related to organization of chromosomes in distinct domains (isochores). Isochore domains differ in gene expression levels, replication timing, levels of meiotic recombination and chromatin structure. The molecular basis for these differences is poorly understood.

**Results:**

We have compared GC- and AT-rich isochores of yeast with respect to chromatin conformation, histone modification status and transcription. Using 3C analysis we show that, along chromosome III, GC-rich isochores have a chromatin structure that is characterized by lower chromatin interaction frequencies compared to AT-rich isochores, which may point to a more extended chromatin conformation. In addition, we find that throughout the genome, GC-rich and AT-rich genes display distinct levels of histone modifications. Interestingly, elimination of the histone deacetylase Rpd3p differentially affects conformation of GC- and AT-rich domains. Further, deletion of *RPD3 *activates expression of GC-rich genes more strongly than AT-rich genes. Analyses of effects of the histone deacetylase inhibitor trichostatin A, global patterns of Rpd3p binding and effects of deletion of *RPD3 *on histone H4 acetylation confirmed that conformation and activity of GC-rich chromatin are more sensitive to Rpd3p-mediated deacetylation than AT-rich chromatin.

**Conclusion:**

We find that GC-rich and AT-rich chromatin domains display distinct chromatin conformations and are marked by distinct patterns of histone modifications. We identified the histone deacetylase Rpd3p as an attenuator of these base composition-dependent differences in chromatin status. We propose that GC-rich chromatin domains tend to occur in a more active conformation and that Rpd3p activity represses this propensity throughout the genome.

## Abstract 2

GC-rich and AT-rich chromatin domains display distinct chromatin conformations and are marked by distinct patterns of histone modifications, and the histone deacetylase Rpd3p is an attenuator of these differences.

## Background

Chromosomes are characterized by regions that differ in base composition [1,2]. These so-called isochores correspond to functionally distinct domains that are cytologically visible as R- and G-bands [2-4]. Functional differences between the two types of regions include higher and lower levels of transcription and meiotic recombination and earlier and later firing of replication origins.

Isochores in the yeast *Saccharomyces cerevisiae *range in size from 5-90 kb [5-9]. Clear evidence that isochores are correlated with functional domains comes from studies of meiotic phenomena in yeast. Programmed double strand break formation and loading of axial structure proteins are much more prominent in GC-rich isochores [7,8,10]. Moreover, when a meiotic recombination hotspot from a GC-rich isochore is inserted into an AT-rich isochore domain, the site adopts the lower recombination activity characteristic of its new environment [11]. This important experiment implies that isochores exert domain-wide control over genes and elements located within them.

GC- and AT-rich isochores differ in chromatin structure, with more open and more compact chromatin in the two types of regions, respectively [12,13]. Additionally, studies of yeast isochores by 3C (chromosome conformation capture) analysis have revealed important structural differences [14]. Chromatin in AT-rich isochores has a longer apparent persistence length than that in GC-rich isochores, suggesting that AT-rich chromatin is less flexible than GC-rich chromatin.

A key feature that affects conformation and activity of chromatin is the histone modification state. For example, telomeres and sub-telomeric regions are regulated by distinct histone deacetylases, Sir2p and Hda1p, respectively [15,16]. However, very little is known about the underlying features that control isochores. Up to now no factors have been identified that act in an isochore-dependent fashion along chromosome arms.

Here, we present evidence that suggests that GC-rich chromatin is in a more extended conformation than AT-rich chromatin and that GC-rich genes on average tend to be more active, thereby extending the analogies between yeast and mammalian isochores. Interestingly, we find that GC-rich and AT-rich regions are marked by distinct levels of a subset of histone modifications. We then show that the histone deacetylase Rpd3p has a novel, base composition-dependent effect on chromatin conformation and gene expression. Comparisons between wild-type and *rpd3Δ *mutant cells with respect to chromatin conformation and transcriptional activity, combined with analysis of the Rpd3p binding pattern in the wild type, led to a model that Rpd3p-dependent histone deacetylation of GC-rich genes directly promotes a more compact chromatin conformation, with a corresponding effect on transcription. We propose that Rpd3p activity attenuates more active GC-rich chromatin throughout the genome.

## Results

### GC-rich isochores have a more extended chromatin conformation than AT-rich isochores

We analyzed conformation of GC- and AT-rich isochores along yeast chromosome III using the 3C methodology. 3C is used to detect the relative frequencies of interaction for different pairs of genomic loci. 3C data can be used to determine the overall spatial conformation of chromosomes and chromosomal sub-domains [14,17-20]. This approach, as previously described in detail [21-23], involves three steps. First, formaldehyde cross-linking is used to trap pairs of interacting chromatin segments (via protein/protein/DNA cross-links). Second, cross-linked chromatin is solubilized and then digested and ligated at low concentration so that cross-linked segments will be preferentially joined. Third, ligation products are detected and quantified by PCR using pairs of primers specific to each pair of interacting loci. Relative levels of different PCR products correspond to the relative interaction frequencies of the various locus pairs.

**Figure 1:**
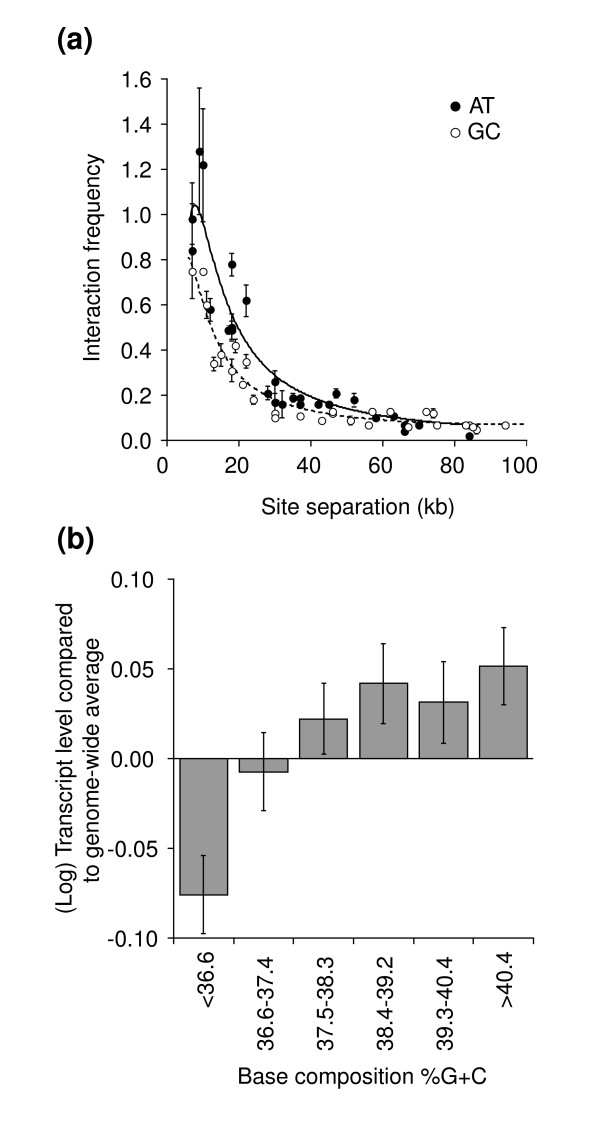
Isochore domains along chromosome III differ in conformation and activity. **(a) **Interaction frequencies (the average of three measurements) between loci located within the AT-rich isochore (positions 100-190 kb) of chromosome III (filled circles) or within the GC-rich isochore domain on the right arm of chromosome III (positions 190-280 kb; open circles) were determined in G1-arrested wild-type cells and plotted against genomic distance that separates each pair of loci. Error bars are standard error of the mean (SEM). Dotted and solid lines indicate fits of the data to equation 1 (Table 1). **(b) **Yeast genes were grouped in six groups dependent on the average base composition of the 4 kb region centered on the start site of the gene is (see Materials and methods). For each group the average steady state transcript level in wild-type cells was determined using data obtained by Bernstein *et al*. [30]. The genome-wide average transcript level was set at zero. The difference between the most GC-rich group and the most AT-rich group is statistically significant (*P *< 0.001). Error bars indicate SEM.

We chose to analyze isochore domains along chromosome III because of their relatively large size (up to 90 kb), which allows detailed 3C analysis. Our previous analysis of these isochores revealed structural differences but did not address whether these differences in interaction frequencies could reflect differences in chromatin compaction [14]. Here we addressed this issue in detail. Nuclei were isolated from alpha-factor arrested (G1) haploid wild-type yeast cells and 3C was performed. Interaction frequencies for pairs of sites located within the GC- and AT-rich domains along the right arm of chromosome III (positions 100-190 kb and 190-280 kb, respectively) were measured. When these frequencies are plotted against the distance between the loci of each pair (the genomic site separation) an inverse relationship between interaction frequency and genomic distance is observed. Moreover, sites located in the GC-rich isochore domain interact less frequently than sites located in the AT-rich isochore domain (Figure 1a).

We next determined whether the difference in interaction frequencies was simply due to lower levels of formaldehyde cross-linking in the GC-rich isochore compared to the AT-rich isochore. We reasoned that formaldehyde cross-linking during the 3C procedure would reduce restriction enzyme digestion efficiency due to cross-linking of proteins to restriction sites and that any differences in cross-linking in GC- and AT-rich domains should be detectable as differences in their susceptibilities to restriction enzyme digestion. We first used a PCR based method that detects partially digested chromatin to confirm that digestion efficiency is inversely proportional to the level of cross-linking (Additional data file 2). We then assessed the digestion efficiencies for several sites located in the GC-rich and AT-rich regions. The fraction of protected restriction sites, and thus the level of cross-linking, in the GC-rich regions was slightly higher than, but not significantly different from, that observed in the AT-rich domain (Additional data file 1 and 2). Similar previous 3C analyses have also shown that digestion and cross-linking efficiency is relatively constant throughout large chromosomal regions [19,24,25]. These results imply that the two types of domains have undergone very similar levels of cross-linking and thus that the difference in interaction frequencies in GC- and AT-rich domains as detected by 3C reflects a difference in spatial conformation.

Interaction frequencies are proportional to the local concentration of the loci and, therefore, differences in interaction frequencies within the GC-rich and AT-rich domains are most straightforwardly attributable to a difference in effective volume between these domains, with the GC-rich isochore occupying a larger volume per kb of DNA (that is, being less compact).

Further details of differences in compaction between GC- and AT-rich domains are provided by analysis of 3C data using a suitable polymer model [14,26-28]. The model used here (equation 1) is the same as that used previously [14,26,29], but is slightly re-arranged in order to allow assessment of chromatin compaction by including a parameter *L *that reflects chromatin compaction:

(1) $$\begin{array}{ccc} X(s)=(k\times L^{-3})\times 0.53\times \beta^{-3/2}\times \exp\left(-\frac{2}{\beta^{2}}\right)\times S^{-3}\frac{{\text{nm}}^{\text{3}}\text{mol}}{\text{liter}} & \text{with} & \beta =\frac{s}{S}\times \left(1-\frac{s}{c}\right) \end{array}$$

This model describes chromatin in terms of three key features: flexibility, apparent circularity and level of compaction (expressed in nm/kb). The parameter *s *is the genomic site separation between two loci (in kb) and *X*(*s*) is the interaction frequency. The parameter *S *is the length of the Kuhn's statistical segment in kb, which corresponds to two times the persistence length and is a measure for the flexibility of the chromatin fiber. The parameter *c *is the apparent circle size of the fiber (in kb). In the case of a fiber engaged in an unconstrained random walk, *c *will be infinitely large, in which case *β *equals *s/S*; any other value of *c *implies the presence of constraints on the path of the chromatin fiber. The parameter *k *is the efficiency of cross-linking [14]. Finally, *L *is the contour length (in nm) of 1 kb of chromatin, referred to as the mass density, and is a measure for the level of compaction of the chromatin fiber. Fitting interaction frequencies to equation 1 yields values for *S*, *c *and for [*k *× *L*^-3^]. Values for the individual parameters *k *and *L *cannot be directly obtained from this analysis and the combined parameter [*k *× *L*^-3^] will be referred to as the apparent compaction factor. However, if *k *is known to be constant, as appears to be the case in the present study (above), variations in this combined parameter can be interpreted as differences in the value of *L*.

When interaction frequencies for the GC- and AT-rich domains, from three independent cultures were fitted to equation 1 (Figure 1a; Table 1), significant differences between the two types of domains become apparent. First, chromatin in the GC-rich domain is significantly more flexible than chromatin in the AT-rich domain (that is, *S *is smaller, *P *< 0.05). Second, the GC-rich domain appears to be in a circular conformation, with an apparent circle size of around 200 kb reflecting the presence of constraints on the chromatin path, consistent with our previous findings [14]. For the AT-rich domain, in contrast, we had to assume (for two out of three cultures) that *c *is infinitely large in order to obtain a good fit, implying the apparent lack of such constraints. Third, an approximately three-fold lower value for the apparent compaction factor [*k *× *L*^-3^] was obtained for the GC-rich domain than for the AT-rich domain (*P *< 0.01).

**Table 1:** Analysis of 3C data reveals significant differences between AT- and GC-rich chromatin in wild-type cells as well as significant effects of deletion of *RPD3 *on [*k *× *L*^-3^] in GC-rich chromatin

	Experiment 1				Experiment 2				Experiment 3				Average		
				
	*k *× *L*^-3^	*S*	*c*	*r*^2^	*k *× *L*^-3^	*S*	*c*	*r*^2^	*k *× *L*^-3^	*S*	*c*	*r*^2^	*k *× *L*^-3^	*S*	*c*
	(M^-1 ^nm^-3 ^kb^3^)	(kb)	(kb)		(M^-1 ^nm^-3 ^kb^3^)	(kb)	(kb)		(M^-1 ^nm^-3 ^kb^3^)	(kb)	(kb)		(M^-1 ^nm^-3 ^kb^3^)	(kb)	(kb)
WT-GC	309	3.6	202	0.92	528	4.3	ND	0.71	576	4.9	171	0.96	471 ± 82	4.26 ± 0.38	190
*rpd3Δ*-GC	240	3.7	186	0.9	314	3.9	ND	0.83	171	3.1	155	0.94	241 ± 41	3.6 ± 0.24	171
															
WT-AT	1,026	4.9	ND	0.89	1,425	5.8	ND	0.64	1,256	6.2	738	0.9	1,235 ± 111	5.6 ± 0.38	738
*rpd3Δ*-AT	1,281	5.5	ND	0.9	1,370	5.8	ND	0.74	1,094	5.3	ND	0.82	1,248 ± 81	5.53 ± 0.15	ND

*rpd3Δ*-AT, AT-rich chromatin in *rpd3Δ *cells; *rpd3Δ*-GC, GC-rich chromatin in *rpd3Δ *cells; WT-AT, AT-rich chromatin in wild-type cells; WT-GC, GC-rich chromatin in wild-type cells.

The difference in the value of [*k *× *L*^-3^] for the GC- and AT-rich domain (Table 1) could reflect differences in cross-linking efficiency (*k*) or compaction (*L*). Since no difference in cross-linking efficiency between GC- and AT-rich domains could be detected, this analysis indicates that there is a 2.5-fold difference in the value of *L*^-3 ^(average of three independent yeast cultures) and thus an approximately 1.4-fold difference in the value of *L*. In other words, the contour length of 1 kb of chromatin in the GC-rich isochore region is approximately 40% larger than the contour length of 1 kb of chromatin in the AT-rich isochore.

### GC-rich genes are more highly expressed

We next examined functional differences between GC- and AT-rich isochores by determining the relationship between base composition of genes and their transcriptional activity throughout the genome in wild-type yeast cells. First, genes were divided into categories based on the average base composition of the surrounding 4 kb region (that is, the average base composition of a gene was determined using a 4 kb window centered around the transcription start site). Genes were then divided into six groups, approximately equal in size, based on regional base composition (Figure 1b). Genes located within 30 kb of telomeres were omitted because these genes are under epigenetic control due to their close proximity to telomeric heterochromatin. Excluding such genes, the final dataset comprised 5,568 open reading frames.

Next we determined average steady-state mRNA levels of genes in each group. The transcriptional activity of each gene is known from data obtained by Bernstein *et al*. [30]. Using their dataset, we find that expression levels of individual genes within each group vary widely, but that the most GC-rich genes as a group are, on average, significantly more transcriptionally active than the most AT-rich isochore group (Figure 1b). Previously, Marin *et al*. [31] reported a similar positive correlation between mRNA levels and GC content of genes in yeast.

### GC-rich and AT-rich chromatin domains are marked by different levels of histone acetylation

Histone modifications can affect the conformation of chromatin fibers and are correlated with gene expression (for example, [32-35]). Given the differences in chromatin conformation and transcriptional activity of GC- and AT-rich chromatin domains, we hypothesized that these domains may also display differences in histone modification status. We used a genome-wide dataset of histone modification levels in wild-type yeast cells obtained by Kurdistani *et al*. [36] to determine average histone modification levels of GC- and AT-rich regions.

**Figure 2:**
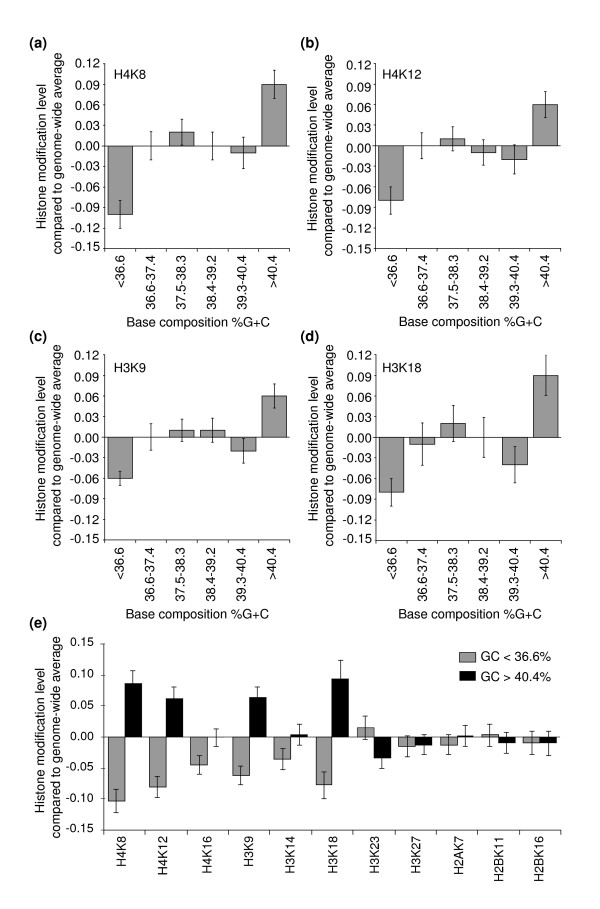
GC-rich and AT-rich genes differ in levels of acetylation of specific histone tail residues in wild-type cells. Genes were grouped in six groups dependent on the average base composition of the 4 kb region centered on the start site of the gene. For each group average levels of acetylation of different histone tail residues were determined using a dataset obtained by Kurdistani and co-workers [36]. **(a-d) **GC-rich genes display higher levels of H4K8, H4K12, H3K9 and H3K18 acetylation compared to AT-rich genes. **(e) **Comparison of the average levels of 11 histone modifications for GC-rich genes (GC > 40.4%) and AT-rich genes (GC < 36.6%). H3 and H4 acetylation is higher for GC-rich genes, whereas H2A and H2B acetylation is not different for the two types of isochore domains.

We again divided all genes into six groups based on their base composition, exactly as described above. For each group we determined the average level of each of 11 histone modifications (Figure 2a-d; Additional data file 3). We found that 4 out of 11 modifications (histone H4 Lys8 (H4K8) and Lys12 (H4K12), and histone H3 Lys9 (H3K9) and Lys18 (H3K18)) are enriched in GC-rich chromatin and depleted in AT-rich chromatin. The levels of the remaining seven modifications were not clearly correlated with base composition (histone H4 Lys16 (H4K16), histone H3 Lys14 (H3K14), LysK23 (H3K23) and Lys27 (H3K27), histone H2A Lys7 (H2AK7), and histone H2B Lys11 (H2BK11) and Lys16 (H2BK16); Additional data file 3). Interestingly, modifications of both H3 and H4 are correlated with base-composition, whereas modifications of H2A and H2B are not. These results demonstrate that GC- and AT-rich chromatin domains display distinct levels of H3 and H4 acetylation (Figure 2e) and provide additional evidence for structural and functional differences of isochore domains in yeast.

### Deletion of *RPD3 *exaggerates the difference in chromatin conformation of GC- and AT-rich domains

The histone deacetylase Rpd3p acts as a repressor of a number of specific target genes throughout the genome [37-39]. In addition, Rpd3p has been shown to affect the global pattern of histone acetylation, over and above its specific effects at target promoters [40]. This global activity is weak, affecting histone acetylation levels only up to two-fold. The significance of these more global weak effects on chromatin structure and gene expression is not well understood. We were interested in the possibility that the global effects of Rpd3p may modulate structural and functional differences between GC- and AT-rich chromatin. To test this, we used 3C to analyze changes in chromatin conformation of GC- and AT-rich domains along chromosome III in an *rpd3Δ *mutant.

**Figure 3:**
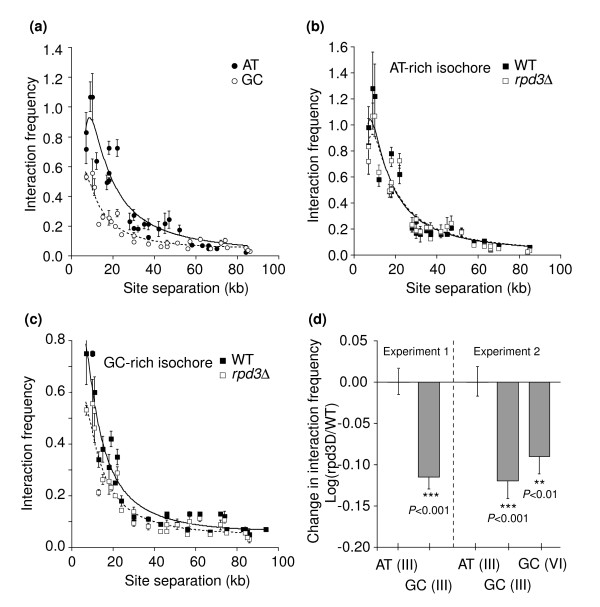
Deletion of *RPD3 *differentially affects conformation of AT- and GC-rich isochore domains. **(a) **Interaction frequencies (the average of three measurements) between loci located within the AT-rich isochore of chromosome III (filled circles) or within the GC-rich isochore domains on the right arm of chromosome III (open circles) were determined in G1-arrested *rpd3Δ *cells. Error bars are standard error of the mean. Dotted and solid lines indicate fits to equation 1 (Table 1). **(b) **Interaction frequencies between loci located in the AT-rich isochore of chromosome III obtained in *rpd3Δ *cells (open squares) and wild type cells (filled squares). Data were normalized such that the average Log of the fold difference between wild-type (WT) cells and *rpd3Δ *cells was zero. Solid and dotted lines indicate fits of the data to equation 1. **(c) **Interaction frequencies between loci located in the GC-rich isochore of the right arm of chromosome III obtained in *rpd3Δ *cells (open squares) and WT cells (filled squares) after normalization. Solid and dotted lines indicate fits of the data to equation 1. **(d) **Interaction frequencies in the GC-rich isochore on the right arm of chromosomes III and VI (GC (III) and GC (VI)) are significantly reduced compared to interaction frequencies in the AT-rich isochore on chromosome III (AT (III)). Data from two biological repeats are shown.

Interaction frequencies between sites located in the GC- and AT-rich isochore domains of chromosome III were determined and plotted against genomic site separation, as described above for wild-type cells (Figure 3a-c). As in the wild type, the GC-rich domain exhibits lower interaction frequencies than the AT-rich domain. However, the magnitude of the difference in interaction frequencies between the two domains is greater in the *rpd3Δ *mutant than in the wild type (compare Figures 3a and 1a; Table 1). This effect can be seen most clearly by normalizing both datasets to the interaction frequencies observed in one of the two domains, for example, the AT-rich domain (see Materials and methods). Such a comparison reveals that all interaction frequencies in the GC-rich domain are approximately 25% lower in the *rpd3Δ *mutant than in the wild type (Figure 3b-d). This effect is statistically significant (*P *< 0.001; Figure 3d) and was observed in three independent *rpd3Δ *cultures (Table 1). We also analyzed a set of interactions along the right arm of chromosome VI, which is characterized by a high GC-content, and found a similar significant decrease in interaction frequencies (Figure 3d).

As discussed above, a difference in interaction frequency between GC- and AT-rich domains could result either from a difference in chromatin compaction or a difference in cross-linking efficiency, and the two possibilities can be distinguished by assessing the efficiency of restriction digestion. When such analysis was performed for *rpd3Δ *cells, we again found, as for wild-type cells, no significant difference in digestion efficiency between GC- and AT-rich isochore domains (Additional data file 2). We conclude that Rpd3p differentially affects the conformation of these GC-rich and AT-rich domains, which results in further exaggeration of their difference in conformation. These observations are important for two reasons. First, they reveal a previously unrecognized base-composition-sensitive effect of this histone deacetylase. Second, they suggest that Rpd3p normally acts to keep the two types of isochore domains from being even more different in conformation than they would otherwise tend to be.

To more fully characterize chromatin conformation in *rpd3Δ *cells, interaction frequencies were fitted to equation 1 (Figure 3a-c; Table 1). Flexibility and apparent circularity of chromatin did not significantly change in *rpd3Δ *cells compared to wild-type cells (Table 1). However, the statistically significant reduction in interaction frequencies in the GC-rich isochore compared to the AT-rich isochore resulted in a five-fold difference in apparent compaction factor [*k *× *L*^-3^] compared to a 2.5-fold difference observed in wild-type cells. Analysis of data from three wild-type cultures and three *rpd3Δ *cultures shows that this effect on the fold difference in [*k *× *L*^-3^] is reproducible and significant (*P *< 0.05). Application of the restriction digestion assay described above further reveals that, as for wild-type cells, this difference is not ascribable to a differential change in efficiency of cross-linking efficiency (*k*) (Additional data file 2).

We conclude that the difference in relative compaction *L *of the chromatin fiber in the GC- and AT-rich isochores has changed in *rpd3Δ *cells. Specifically, in *rpd3Δ *cells, the value of *L *is 1.7-fold higher, and compaction correspondingly lower, in the GC-rich isochore compared to the AT-rich isochore.

### Deletion of *RPD3 *most strongly activates transcription of GC-rich genes

Our results suggest that Rpd3p activity differentially affects GC-rich and AT-rich chromatin. We next tested whether this effect was also reflected in differential modulation of expression of GC- and AT-rich genes throughout the yeast genome. This question was addressed using a genome-wide dataset generated in Tsukiyama's laboratory [37] that describes the effects of deletion of *RPD3 *on transcription throughout the yeast genome.

**Figure 4:**
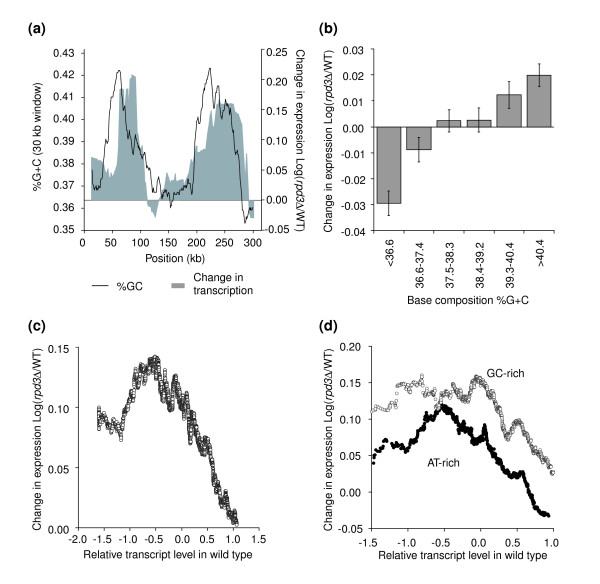
Rpd3p displays base composition-dependent activity. **(a) **Patterns of base composition (line) and gene activation (gray area) in *rpd3Δ *cells along chromosome III as determined by sliding window analysis using a window size of 30 kb and the transcription start sites as midpoints (step size 1 open reading frame). The genome-wide dataset describing the effect of deletion of *RPD3 *was produced by Fazzio *et al*. [37]. **(b) **Genes were grouped in six groups dependent on the average base composition of the 4 kb region centered on the start site of the gene. For each group the average Log of the fold change in transcription in an *rpd3Δ *mutant compared to wild type was calculated. More GC-rich genes are more activated than more AT-rich genes (*P *< 10^-13 ^for the difference between the most GC-rich genes and the most AT-rich genes). **(c) **The moving average (window size 200, step size 1 open reading frame) of the Log of the fold change in transcript level in *rpd3Δ *is plotted against transcript level in wild type. **(d) **A similar analysis as in **(c) **is performed with genes that are in the most GC-rich group and in the most AT-rich group (window size of 100 genes). GC-rich genes are more up-regulated in *rpd3Δ *cells.

First, we examined whether the genes in the relatively large 90 kb GC-rich and AT-rich isochores along chromosome III are differentially affected by deletion of *RPD3*. We calculated the average change in transcription in *rpd3Δ *versus wild-type cells along chromosome III as a function of gene position along the chromosome. Comparison of average base composition with average global change in transcription shows that deletion of *RPD3 *had little effect on transcription of the central AT-rich isochore domain. In contrast, transcription in the GC-rich isochore domains was significantly more increased (Figure 4a; *P *< 0.001).

Next we analyzed whether deletion of *RPD3 *has a general differential effect on expression levels of GC- and AT-rich genes throughout the genome. We calculated the effect of deletion of *RPD3 *on expression of the same six groups of genes with different base compositions as described above (Figure 1b). We found that all six groups exhibit increased average levels of transcription in the *rpd3Δ *mutant (genome-wide average Log(*rpd3Δ*/WT) = 0.08) but that the magnitude of this effect varies in proportion to GC content. More GC-rich genes are significantly more up-regulated in *rpd3Δ *cells than more AT-rich genes (Figure 4b). These data confirm that elimination of Rpd3p affects most regions of the genome [15,38] and, in addition, reveal a previously unappreciated fact that base composition is an important feature in determining the magnitude of this effect.

### The base composition-dependent effect of *rpd3Δ *is independent of gene expression level

To characterize the base-composition sensitive effect of deletion of *RPD3 *in more detail, we analyzed whether it was related to the level of expression of genes in wild-type cells. First, we determined the general relationship between mRNA levels of genes in wild-type cells and the fold change in expression in *rpd3Δ *cells. We found that deletion of *RPD3 *most strongly activated genes that are expressed at relatively low levels in wild-type cells (Figure 4c), as expected for deletion of a transcriptional repressor. Next we analyzed whether this relationship is different for GC- and AT-rich genes. Interestingly, for both the most GC-rich and AT-rich groups of genes we found a similar negative correlation between transcript level in wild-type cells and increase in transcription in *rpd3Δ *cells. Importantly, however, for all levels of transcription, GC-rich genes are more up-regulated upon deletion of *RPD3 *than AT-rich genes that are expressed at similar levels in wild-type cells (Figure 4d). These observations reveal that Rpd3p mediates transcriptional control via two independent effects. At one level, Rpd3p-mediated inhibition is correlated with steady-state expression levels of genes. At the second level, Rpd3p inhibits transcription in a GC content-dependent manner. The GC content-dependent activity is not correlated with the steady-state expression level of genes.

**Figure 5:**
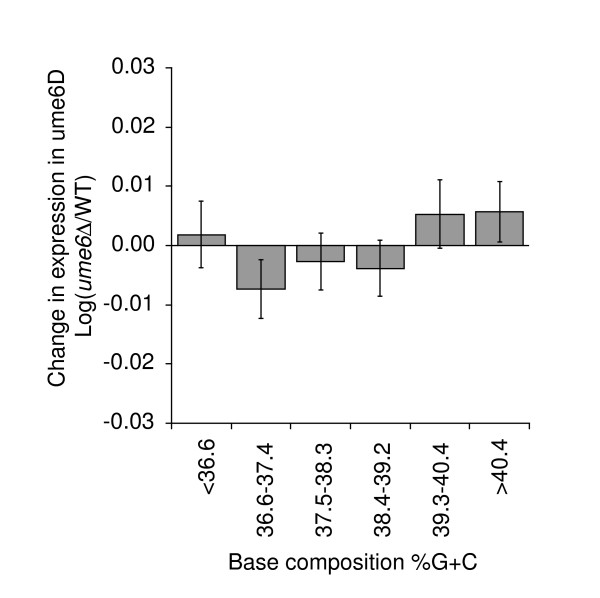
Deletion of *UME6 *does not differentially affect GC- and AT-rich genes. Average change in gene expression levels in *ume6Δ *cells compared to wild type for each of the six groups of genes with increasing GC content. Expression data are from Fazzio *et al*. [37].

These observations suggest that the base composition-dependent activity of Rpd3p is not dependent on local and gene-specific control of promoter activity, but instead may be related to more general features of chromatin conformation in GC-rich regions of the genome. In that case, we predict that the base composition-dependent activity of Rpd3p will be independent of local targeting to specific target genes. To test this we analyzed the effects of deletion of *UME6*. Ume6p recruits Rpd3p to many of its specific target promoters and the effects of deletion of *UME6 *display many similarities to those observed upon deletion of *RPD3 *[41]. We used a dataset obtained by Fazzio *et al*. [37] to determine whether deletion of *UME6 *differentially affects GC- and AT-rich genes. Interestingly, we did not find significant base composition-dependent changes in gene expression (Figure 5). Therefore, Ume6p-dependent recruitment does not appear to be involved in base composition-dependent activity of Rpd3p. We propose that the non-targeted global activity of Rpd3p affects transcription and chromatin conformation in a base composition-dependent manner.

### Rpd3p binding and Rpd3p-mediated histone deacetylation are stronger for GC-rich genes

**Figure 6:**
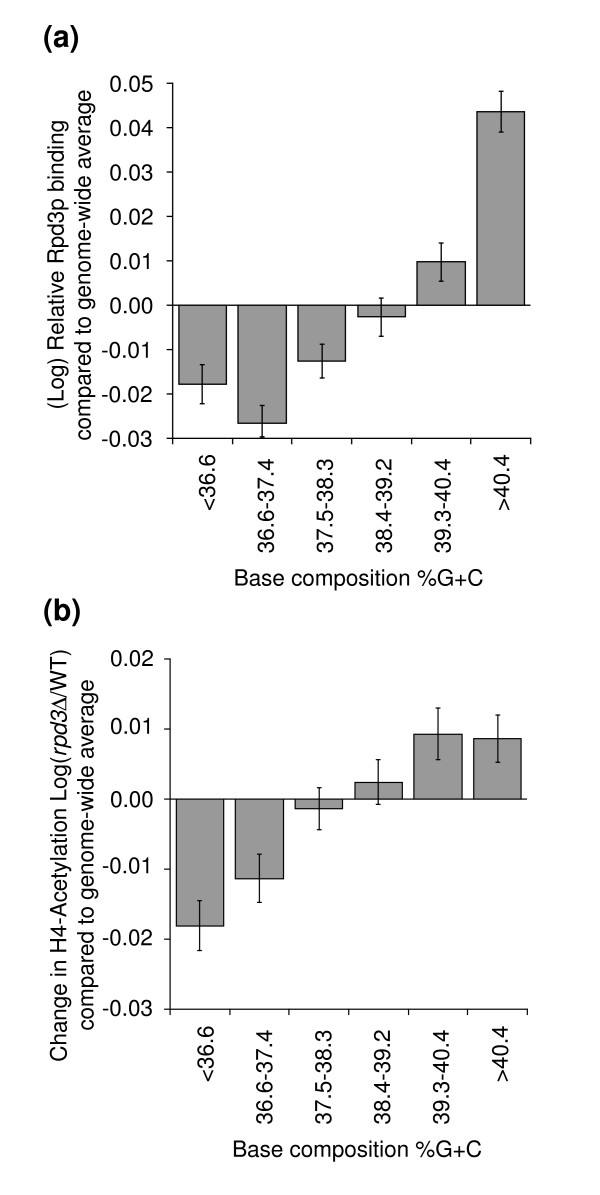
Rpd3p binding in wild-type and histone acetylation in *rpd3Δ *cells in AT-rich and GC-rich isochors. **(a) **Average levels of Rpd3p binding to each of the six groups of genes with increasing GC content. Rpd3p binding data are from Humphrey *et al*. [42]. **(b) **Average change in H4 acetylation of the upstream region of each of the six groups of genes with increasing GC content. Acetylation data were obtained by Bernstein *et al*. [30].

To determine whether the base composition-dependent effects of Rpd3p are direct and not due to indirect effects of altered expression of a downstream target gene, we analyzed the patterns of Rpd3p binding and Rpd3p-mediated histone H4 deacetylation. Relative levels of Rpd3p-binding throughout the yeast genome have been determined by Humphrey *et al*. [42]. Using these data, we determined the relative average levels of Rpd3p binding to genes in each of the six base-composition-based groups defined above (Figure 1b). We found that the level of bound Rpd3p is significantly higher for the most GC-rich genes than for the rest of the genome (*P *< 0.01; Figure 6a).

For analysis of Rpd3p-mediated histone H4 acetylation, we employed a dataset of Bernstein *et al*. [30], who analyzed H4 acetylation levels in intergenic regions throughout the genome in wild-type and *rpd3Δ *cells. We found that elimination of Rpd3p increases H4 acetylation of GC-rich genes more strongly than that of AT-rich genes (Figure 6b). These observations imply that Rpd3p binds more strongly to GC-rich genes, resulting in lower levels of histone acetylation and, thereby, directly affects chromatin conformation and expression level of GC-rich genes.

### Base-composition-dependent modulation of gene expression requires histone deacetylase activity and is specific for Rpd3p

**Figure 7:**
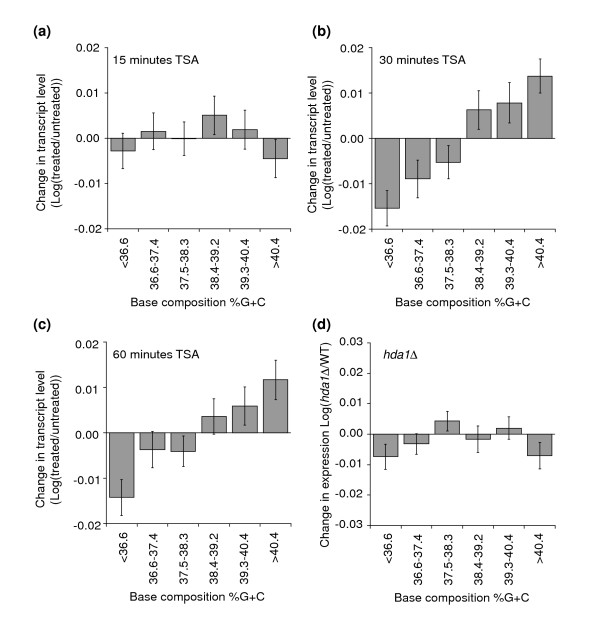
Inhibition of the histone deacetylase activity of Rpd3p results in base composition-dependent gene activation. The dataset describing changes in gene expression upon TSA treatment was obtained from Bernstein *et al*. [41]. Genes were divided into six groups dependent upon their base composition (as in Figure 1b) and per group the average change in gene expression upon TSA treatment was determined. Error bars are standard error of the mean. Cells were treated for **(a) **15 minutes, **(b) **30 minutes and **(c) **60 minutes with TSA. **(d) **Average change in gene expression levels in *hda1Δ *cells for each of the six groups of genes with increasing GC content. Expression data are from Bernstein *et al*. [41].

To determine whether the base composition-dependent effect of *rpd3Δ *is due to loss of histone deacetylase activity, we investigated the effect of treatment with the histone deacetylase inhibitor trichostatin A (TSA) on expression of GC- and AT-rich genes in wild-type cells. Bernstein and co-workers [41] have analyzed genome-wide changes in gene expression at various time points after addition of TSA. We have analyzed their data in the same way as described above to determine whether TSA treatment differentially affects expression of GC-rich and AT-rich genes. We found that after 30 and 60 minutes of exposure to TSA, GC-rich genes are more activated than AT-rich genes, whereas no such effect was observed after 15 minutes (Figure 7a-c). This result confirms that histone deacetylation plays an important role in differentially modulating GC- and AT-rich genes. We do note that the base composition-dependent effect of TSA treatment occurs more slowly than up-regulation of specific Rpd3p target genes, which is already observed after 15 minutes of TSA treatment [41].

TSA inhibits not only Rpd3p, but also another globally acting histone deacetylase, Hda1p. Therefore, to determine whether the base composition-dependent effect of TSA treatment is a result of inhibition of Rpd3p as well as Hda1p, or is specifically due to inhibition of Rpd3p only, we analyzed the effects of deletion of *HDA1*. Using an expression dataset obtained by Bernstein *et al*. [41], we found that deletion of *HDA1 *does not result in base composition-dependent changes in gene expression (Figure 7d). This result suggests that base-composition sensitive activity is specific for the histone deacetylase activity of Rpd3p.

## Discussion

We show that yeast isochores share characteristics with those found in higher eukaryotes in addition to those described before. Our results indicate that GC-rich and AT-rich domains are both structurally and functionally distinct. First, interaction frequencies within GC-rich chromatin tend to be lower than those in AT-rich chromatin, which is in agreement with a more extended chromatin conformation, as observed in higher eukaryotes [12,13]. Second, similar to mammalian isochores, genes located in the most GC-rich regions of the yeast genome are, on average, more highly expressed (for example, [4]). Importantly, we found that GC-rich genes display higher levels of H3 and H4 acetylation compared to more AT-rich genes. Finally, we identify Rpd3p as a molecular component involved in base composition-dependent control of chromatin structure and function. This role of Rpd3p may be conserved in higher eukaryotes as it is also associated with less condensed interbands in *Drosophila *[43]. This activity appears to be specific for Rpd3p as we did not detect a base composition-dependent activity of another globally acting histone deacetylase, Hda1p.

Rpd3p has been shown to have two distinct modes of action. First, Rpd3p is recruited to specific target genes to modulate their expression. Second, Rpd3p acts in a global and non-targeted fashion to deacetylate bulk chromatin. We propose that the base composition-dependent effects of Rpd3p are related to its global activities. First, the magnitudes of these GC-content dependent effects are subtle, similar to the previously described effects of Rpd3p on global histone acetylation [40]. Second, deletion of Ume6p, a protein involved in recruitment of Rpd3p to many of its specific target genes [38], does not result in up-regulation of GC-rich genes, indicating that Rpd3p interacts with GC-rich genes in a Ume6p-independent manner. Third, the GC content-dependent effects are not correlated with the steady-state expression of genes, and thus seem unrelated to local promoter controls.

We favor the model that the global and untargeted activity of Rpd3p acts predominantly and/or has the largest effect on GC-rich chromatin. First, Rpd3p binds GC-rich genes more prominently than AT-rich genes. Second, deletion of *RPD3 *results in increased H4 acetylation, particularly of GC-rich genes. Finally, treatment of wild-type cells with the histone deacetylase inhibitor TSA activates GC-rich genes more strongly than AT-rich genes. However, we did observe that TSA induced activation of GC-rich genes requires more time than induction of many direct target Rpd3p genes. This relatively slow effect could be interpreted to mean that the base composition-dependent effects of deletion of *RPD3 *are indirect and are due to altered expression of a specific Rpd3p target gene that, in turn, encodes a protein that acts in a GC content-dependent fashion. Alternatively, and consistent with the Rpd3p localization and acetylation data, Rpd3p does directly affect expression of GC-rich genes, but this more global and non-targeted process occurs at a longer time scale or requires passage through a specific phase of the cell cycle.

An alternative or additional molecular explanation of the observed phenomena is related to potential base composition-dependent differences in wrapping of DNA around histones. AT-rich DNA may be more flexibly and more easily wrapped around nucleosomes than GC-rich DNA [44]. This physical model implies intrinsic differences in nucleosome organization dependent on base composition and does not require that histone modifying enzymes act in a base composition-dependent fashion *per se*. In this model, histone modifying enzymes recognize differences in intrinsic conformation of GC- and AT-rich chromatin. Rpd3p may preferentially act on the nucleosome organization of GC-rich chromatin. Similarly, acetyl transferases may preferentially modify GC-rich domains in wild type, resulting in higher levels of histone H3 and H4 acetylation, as we observed here. Based on these considerations, we predict the presence of histone acetyl transferases that act most prefentially on GC-rich chromatin.

In light of these observations, we can interpret our 3C analysis more precisely. The 3C results show that deletion of *RPD3 *differentially affected the conformation of GC- and AT-rich isochore domains along chromosome III, but did not allow determination of which of the two types of domains, or both, displayed an altered conformation. When Rpd3p activity affects GC-rich genes most prominently, the most parsimonious explanation of our 3C data is that deletion of *RPD3 *most strongly affects the conformation of the GC-rich domain, resulting in a more extended and transcriptionally active chromatin conformation, consistent with predicted relationships between transcription, histone acetylation and chromatin conformation.

GC-rich chromatin displays lower interaction frequencies, as detected by 3C, than AT-rich chromatin. Analysis of cross-linking efficiency suggests that both types of domains are cross-linked with similar frequencies (Additional data file 2) and, therefore, have similar protein densities. Histones are the most abundant chromatin proteins, and thus our results suggest that GC-rich and AT-rich regions have similar levels of histone binding. Consistent with this hypothesis, Nagy *et al*. [45] found no correlation between base composition and regions depleted in nucleosomes. Previously, we found a decrease in interaction frequencies upon activation of the *FMR1 *gene in human cells [19], similar to the observed changes in *rpd3Δ *cells described here, suggesting that reduced 3C interaction frequencies may be a general characteristic of active chromatin.

The base composition-dependent effect of Rpd3p activity affects expression of genes independent of their steady state level of expression. Genes with the same steady state expression level in wild type are more strongly repressed by Rpd3p when they are GC-rich than when they are AT-rich. This implies that GC-rich genes are intrinsically more active, consistent with higher steady state levels of H3 and H4 acetylation, as we observed here, and that Rpd3p acts as an attenuator of these genes. Based on these considerations, we propose that chromatin status is regulated through a homeostatic and highly dynamic mechanism involving counteracting activating and repressing activities. A similar model of dynamic global acetylation and deacetylation has been proposed by Katan-Khaykovich and Struhl [46] and by Clayton *et al*. [47].

## Conclusion

The findings described here uncover novel GC content-dependent differences in chromatin conformation, regulation, histone modification status and transcription. These findings are significant from four perspectives. First, they provide new information about the nature and functional significance of base composition variation along chromosomes. Second, they show that GC-rich chromatin is most acetylated and is attenuated by the histone deacetylase Rpd3p, thus providing a first clue as to how regions with different base-compositions are differentially regulated. Third, they show that the roles of Rpd3p can be separated into independent components, with a GC-content-dependent global activity layered on top of its targeted and gene specific effects. Finally, they suggest that functional and structural differences between GC- and AT-rich regions are determined in part by balances between activating and repressing activities.

## Materials and methods

### Strains

Yeast strains were derived from SK1. The genotype of the wild-type strain (NKY2997) is: *MATa*, *ho::LYS2*, *lys2*, *ura3*, *nuc1::LEU2*. The genotype of the *rpd3Δ *strain (JDY172) is *MATa*, *ho::LYS2*, *lys2*, *ura3*, *nuc1::LEU2*, *rpd3::KanMX4*. The *RPD3 *gene was deleted using a standard PCR based gene disruption strategy [48].

### 3C analysis

Nuclei were isolated from G1-arrested cells and 3C analysis was performed as described previously [14]. A control PCR template was generated by digestion and random ligation of purified yeast genomic DNA (NKY2997). All templates were titrated to determine the linear range of PCR. PCR products were quantified on 1.5% agarose gels in the presence of ethidium bromide. Interaction frequencies were calculated in triplicate by determination of the ratio of the amount of PCR product obtained with the 3C template divided by the amount of PCR product obtained with the control template [14]. Reproducibility was confirmed by analysis of three independent wild-type cultures and three *rpd3Δ *cultures. Data from wild-type cells and *rpd3Δ *cells was normalized so that the average Log of the fold difference in interaction frequencies in *rpd3Δ *cells compared to wild-type cells for loci in the AT-rich domain of chromosome III was zero.

### Sliding window analysis and generation of isochore groups

The chromosomal position of each open reading frame was defined as its 5' start site. Base composition was determined using EMBOSS Isochore [49] with a window size of 4 kb and a step size of 1 kb. As a result, base composition values are assigned every 1 kb. To determine the regional base composition of each gene, the base composition value determined for the position closest to its 5' start site was chosen.

### Analysis of microarray data

For each open reading frame the fold change in transcription in an *rpd3Δ *mutant compared to wild type was obtained from a dataset generated by Fazzio and co-workers [37] and log-transformed. Sliding window analysis was used to plot regional effects of deletion of *RPD3 *along chromosomes III (Figure 4a). A window of 30 kb was selected. The step size was 1 open reading frame.

Steady-state transcription levels in exponentially growing wild-type cells were obtained by Bernstein and co-workers [30]. Histone acetylation levels in wild-type cells were determined by Kurdistani and co-workers [36]. Rpd3p binding to intergenic regions was determined using data obtained by Humphrey and co-workers [42]. The change in the level of histone H4 acetylation of intergenic regions in *rpd3Δ *cells was determined using data obtained by Bernstein *et al*. [30]. Rpd3p binding and change in acetylation levels were assigned to a downstream open reading frame as described in [30,42]. Datasets describing the effect of TSA treatment on gene expression were obtained by Bernstein *et al*. [41]. Data were log-transformed and zero-centered.

## Additional data files

The following additional data are available with the online version of this paper. Additional data file 1 describes the methodology used to quantify cross-linking efficiency in GC- and AT-rich domain in wild-type and *rpd3Δ *cells. Additional data file 2 is a figure showing the relationship between the level of cross-linking and the efficiency with which chromatin is digested. This figure also displays the digestion efficiencies of GC- and AT-rich chromatin in wild-type and *rpd3Δ *cells. Additional data file 3 displays the level of seven histone modifications in relation to base composition.

## Supplementary Material

Additional data file 1 Methodology used to quantify cross-linking efficiency in GC- and AT-rich domains in wild-type and *rpd3Δ *cells. Click here for file

Additional data file 2 Relationship between the level of cross-linking and the efficiency with which chromatin is digested and the digestion efficiencies of GC- and AT-rich chromatin in wild-type and *rpd3Δ *cells. For details see Additional data file 1. Click here for file

Additional data file 3 Level of seven histone modifications in relation to base composition. Click here for file
