# Supplementary material for: GC- and AT-rich chromatin domains differ in conformation and histone modification status and are differentially modulated by Rpd3p
Source: Genome Biol. 2007 Jun 18;8(6):R116. doi: 10.1186/gb-2007-8-6-r116 (PMC2394764; doi:10.1186/gb-2007-8-6-r116)
Supplement: Additional data file 1 — Methodology used to quantify cross-linking efficiency in GC- and AT-rich domains in wild-type and rpd3Δ cells. [file gb-2007-8-6-r116-S1.doc]

Supplemental Online Material

Determination of digestion efficiency

We reasoned that cross-linking efficiency could be measured by determination of the efficiency by which cross-linked chromatin can be digested with a restriction enzyme. An assay for analysis of restriction digestion efficiency during 3C analysis is described in Additional file 2. When digestion is complete, cross-linking and ligation creates a single ligation product (Additional file 2 panel A, ligation product 1). In contrast, if digestion is not complete, a larger ligation product can arise which includes an adjacent restriction fragment (Additional file 2 panel A, ligation product 2). The two species can be detected as corresponding PCR products. The ratio of the amount of the larger product and the smaller product equals the fraction of restriction sites that is not digested. The difference in molecular weight of the two PCR products, which affects ethidium bromide staining, is corrected for by dividing the ratio of the amounts of the products by the ratio of the size of the products. The larger PCR product is most reliably detected when its size is only marginally larger than the smaller PCR product. Thus the size of the adjacent restriction fragment (black bar in Additional file 2 panel A) ideally should be only 50-100 basepairs, which limits the number of sites for which digestion efficiency can accurately be determined. Digestion efficiency of each site is typically determined based on 6 measurements.

It is important to note that this digestion efficiency assay is distinct from the typical nuclease sensitivity assays that are widely used to determine the level of chromatin accessibility. Here, digestion efficiency is determined after solubilization of cross-linked chromatin in an SDS containing buffer so that all non-cross-linked proteins are removed from the DNA and thus this assay solely detects the level of protein cross-linked to DNA [14].

Using this assay, we could confirm that digestion efficiency is inversely and linearly related to the efficiency of formaldehyde induced cross-linking. First, nuclei were isolated from G1 arrested wild type and *rpd3Δ* cultures and subjected to 3C analysis as described [14] using 0%, 0.2%, 0.4%, 0.8% and 1% formaldehyde. DNA concentrations were determined using ethidium-containing agarose gels. Equal amounts of each DNA sample were used as PCR template to determine cross-linking frequencies and digestion efficiencies using a pair of primers on chromosome III that can detect the interaction between two *Eco*RI fragments separated by 19 kb. Digestion efficiency and interaction frequency of the two loci were determined as a function of increasing formaldehyde concentrations. As expected, the fraction of sites protected from digestion and the frequency with which the two loci could be cross-linked both increased with increasing formaldehyde concentration (Additional file 2 panel B). Importantly, an apparent linear relationship between these parameters was found. Similar linear relationships between interaction frequencies and digestion efficiencies were observed for sites separated by 21, 52 and 106 kb (not shown). This analysis shows that digestion efficiency directly reports the efficiency of formaldehyde induced cross-linking.

We next used this assay to determine the efficiency of restriction enzyme digestion in the GC- and AT-rich isochore domains that had been subjected to cross-linking under standard 3C conditions**.** In the experiment shown, absolute digestion efficiency in *rpd3Δ* cells was slightly higher than in wild type cells (compare Additional file 2 panel D with panel C), but this difference was not reproducible as in repeated experiments we typically did not observe a difference between wild type and *rpd3Δ* cells. By fitting interaction data to Eq. 1, we found a 3-fold difference in the apparent compaction factor [*k* x *L^-3^* ] in wild type cells and a 5-fold difference in *rpd3Δ* cells (see Table 1). When these 3- and 5-fold differences are solely due to differences in cross-linking efficiency we would predict to find 3- and 5-fold lower fractions of protected restriction sites in the GC-rich domains compared to the AT-rich domain. In contrast, we do not find significant differences in digestion efficiency. We conclude that both in wild type cells and in *rpd3Δ* cells cross-linking efficiency in these AT- and GC-rich isochore domains is not significantly different.

**Legend Additional file 2.**

**Determination of cross-linking efficiency by quantification of restriction digestion efficiency**

**A.** Partial digestion of a restriction site during the 3C experiment gives rise to two different ligation products that can be distinguished by size. **B.** Digestion efficiency and interaction frequency of two loci on chromosome III was determined for a range of formaldehyde concentrations (indicated by percentages in the graph). A linear relationship between digestion efficiency and apparent interaction frequency was found. **C.** Digestion efficiencies for 5 sites in the AT-rich isochore of chromosome III and 6 sites in the GC-rich isochores on chromosome III and VI were determined. Mean and standard error of the mean are shown. No difference in digestion efficiency was observed indicating that formaldehyde cross-linking is very similar in AT- and GC-rich isochore domains. **D.** Digestion efficiency in AT- and GC-rich isochore domains in *rpd3Δ* cells.

**Legend Additional file 3**

Average levels of acetylation of specific histone tail residues in wild type cells in relation to base-composition

Genes were grouped in 6 groups dependent on the average base composition of the 4 kb region centered on the start site of the gene (as in Fig. 1B). For each group average levels of acetylation of different histone tail residues was determined using a dataset obtained by Kurdistani and co-workers [36]. The 7 modifications shown here do not appear to be enriched or depleted in GC-rich and AT-rich chromatin domains.
